# Regeneration and repair of human digits and limbs: fact and fiction

**DOI:** 10.1002/reg2.41

**Published:** 2015-10-13

**Authors:** Shyh‐Jou Shieh, Tsun‐Chih Cheng

**Affiliations:** ^1^ Division of Plastic and Reconstructive Surgery, Department of Surgery, National Cheng Kung University Hospital, College of MedicineNational Cheng Kung UniversityTainanTaiwan; ^2^ International Research Center for Wound Repair and Regeneration (iWRR)National Cheng Kung UniversityTainanTaiwan

**Keywords:** Digit and limb regeneration, digit and limb surgical reconstruction, regenerative medicine, regrowing human digits and limbs, tissue engineering

## Abstract

A variety of digit and limb repair and reconstruction methods have been used in different clinical settings, but regeneration remains an item on every plastic surgeon's “wish list.” Although surgical salvage techniques are continually being improved, unreplantable digits and limbs are still abundant. We comprehensively review the structural and functional salvage methods in clinical practice, from the peeling injuries of small distal fingertips to multisegmented amputated limbs, and the developmental and tissue engineering approaches for regenerating human digits and limbs in the laboratory. Although surgical techniques have forged ahead, there are still situations in which digits and limbs are unreplantable. Advances in the field are delineated, and the regeneration processes of salamander limbs, lizard tails, and mouse digits and each component of tissue engineering approaches for digit‐ and limb‐building are discussed. Although the current technology is promising, there are many challenges in human digit and limb regeneration. We hope this review inspires research on the critical gap between clinical and basic science, and leads to more sophisticated digit and limb loss rescue and regeneration innovations.

## Introduction

Numerous tissue defects that need repair and reconstruction are the daily fare of plastic and reconstructive surgeons. Tissue loss negatively affects patients who have congenital anomalies or undergo tumor‐lesion resection or trauma injuries. Although the success rates of surgical repair and reconstruction of human digit and limb injuries have climbed in step with the technological advances in modern surgical instruments, microscopes, and surgical techniques, some stubbornly irreparable and unreplantable digit and limb injuries remain. Composite tissue allotransplantation for patients with an amputated hand is a controversial alternative but increasingly being adopted worldwide; however, the benefits must be carefully weighed against the risks of lifelong immunosuppressive therapy (Shores et al. 2015; Alolabi et al. 2015). Cosmetic and functional prostheses are important aids and another choice for major upper‐limb amputations. However, although most patients are satisfied with their prostheses and their utility, and with their good prosthetic skills, they actually do not use their prostheses for more than about half of the activities of daily living (Ostlie et al. 2012). Hence, to deal with this clinical issue, scientists propose creating digits and limbs in the laboratory using the regenerative approaches of developmental biology and tissue engineering.

Digital phalanx regeneration after amputation in humans has been observed and reported in the clinical literature (Mennen & Wiese 1993; Vidal & Dickson 1993; Lee et al. 1995). However, this is more frequently seen in young children (Illingworth 1974). All successful regeneration cases were treated with direct suturing or covering with a semi‐occlusive or occlusive dressing. This type of management was thought to provide proper environments for fingertip regeneration. However, as the child observed in clinical practice grew older, the regenerative ability of the fingertips decayed. Hypotheses on hindrance to adult digit regeneration include dominant inflammatory reaction rather than regenerative response in adults, tumor suppression gene activation causing progenitor cells’ quiescent tendency in the post‐neonatal period and depletion of progenitor cells with advancing age (Lehoczky et al. 2011). Therefore, scientists are trying to find other solutions, from the point of view of developmental biology, to restore both the morphology and function of the missing digits and limbs. They are trying to determine how regeneration occurs and how it works macroscopically and microscopically. We all hope that digit and limb regeneration can soon be used for humans. Many regeneration models of amphibians and other animals have been identified and elaborately described. However, the capacities of regeneration in different creatures involve unequally distributed cell pluripotentiality. Sánchez Alvarado (2012), one of the experts in this field, deduced that mechanisms for regulating stem cell pluripotentiality increased as morphological complexity increased; therefore, as evolutionary ladders grew higher, animals were increasingly deprived of their potential for regeneration.

Tissue engineering is currently a promising field, and researchers hope to combine specific ex vivo cultured cells together in three‐dimensional biomaterial scaffolds with proper bioactivators to create tissue and build organs in the laboratory (Shieh & Vacanti 2005). A great deal of work has been done to advance the tissue engineering approach to human digit and limb regeneration (Isogai et al. 1999; Vacanti et al. 2001; Wang et al. 2009), but there are still numerous undisclosed obstacles that need to be dealt with. Specifically, digits and limbs contain many different components, such as skin, muscle, tendon, bone, joint, nerves and blood vessels, all of which must be assembled and perfectly integrated to restore functionality.

Digit and limb amputation injury might be seriously disabling or life‐threatening for humans because we lack regeneration ability. Interdisciplinary approaches are currently being used to examine how to improve the possibility of making our species capable of regeneration. Discovering or developing regeneration mechanisms and pathways would have a tremendous effect on the outcomes of certain injuries and diseases. Is regenerating a human digit or limb only a dream, or is it a realistic goal? Scientists are at odds about this.

## Clinical Approach for Tissue or Digit and Limb Loss of the Upper Extremity

Hand trauma is very common, and the severity of the injury varies from simple lacerations, to soft tissue losses with or without bone exposure, to total amputation. The general principles for repairing or reconstructing tissue or digit and limb loss of the upper extremity follow the reconstructive ladder: primary repair, secondary intention healing, skin graft, local or regional flap, and microvascular replantation or free tissue transplantation.

**Figure 1:**
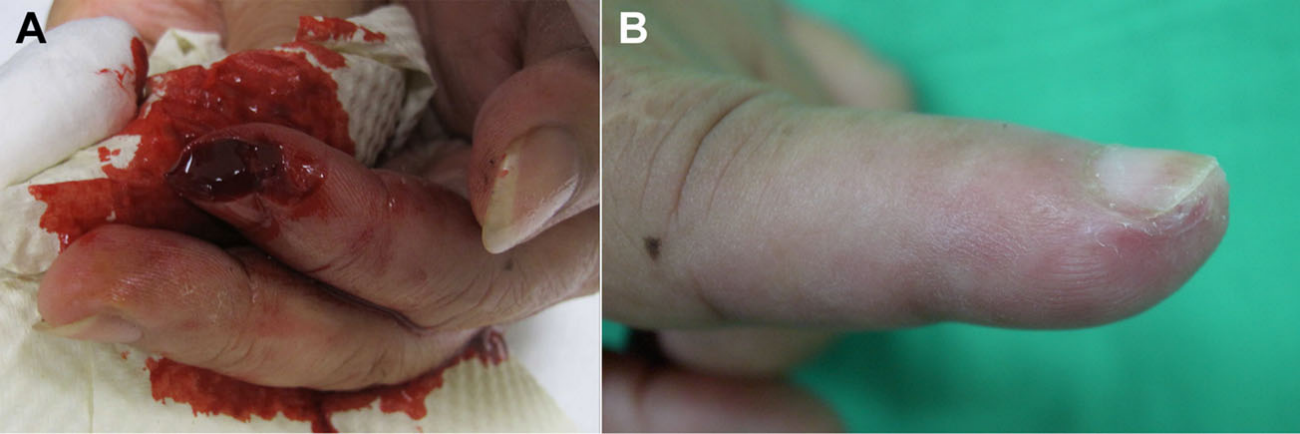
A secondary intention healing. (A) A 1.0 × 0.7 cm skin loss from a cut injury on the left index fingertip. (B) The wound was healed by secondary intention. The sensory recovery and cosmetic result were excellent.

**Figure 2:**
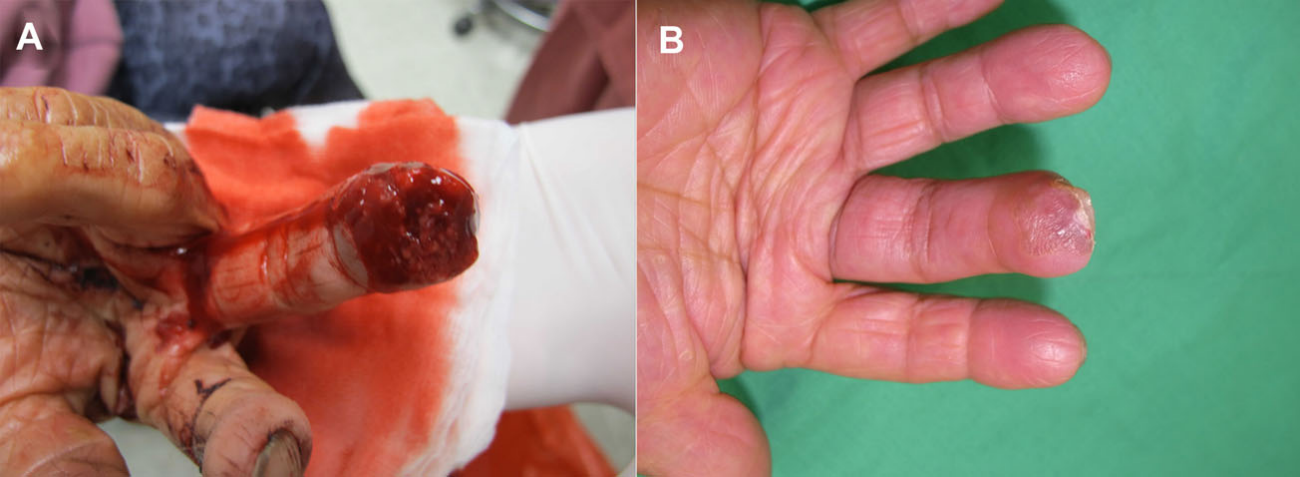
A skin graft. (A) A crush injury with a 1.5 × 1.4 cm skin loss without bone exposure over the right long‐finger pulp. (B) A full‐thickness skin graft from his groin region covered the wound. The digital pulp was totally healed at a 3‐week follow‐up.

For fingertip or distal finger injuries, which are defined as injuries involving the part of the digit distal to a distal interphalangeal joint, the injury assessment should confirm whether it is dorsal or volar (palmar), what the angle is, whether the nails or nail beds are involved, and whether the bone is exposed. Primary repair is always favored if the wound is a simple laceration or only a minimal tissue loss. If there is no bone exposure for a distal digit injury or amputation, secondary intention healing is indicated for wounds less than 1.0−1.5 cm^2^ (Lemmon et al. 2008; Friedrich & Vedder 2011; Tang et al. 2014), which will always result in a sensate fingertip (Fig. 1). However, a skin graft is recommended for wounds more than 1.0−1.5 cm^2^ without bone exposure (Fig. 2). Original skin (if available) or a full‐thickness skin graft is better than a split‐thickness skin graft in such a situation.

**Figure 3:**
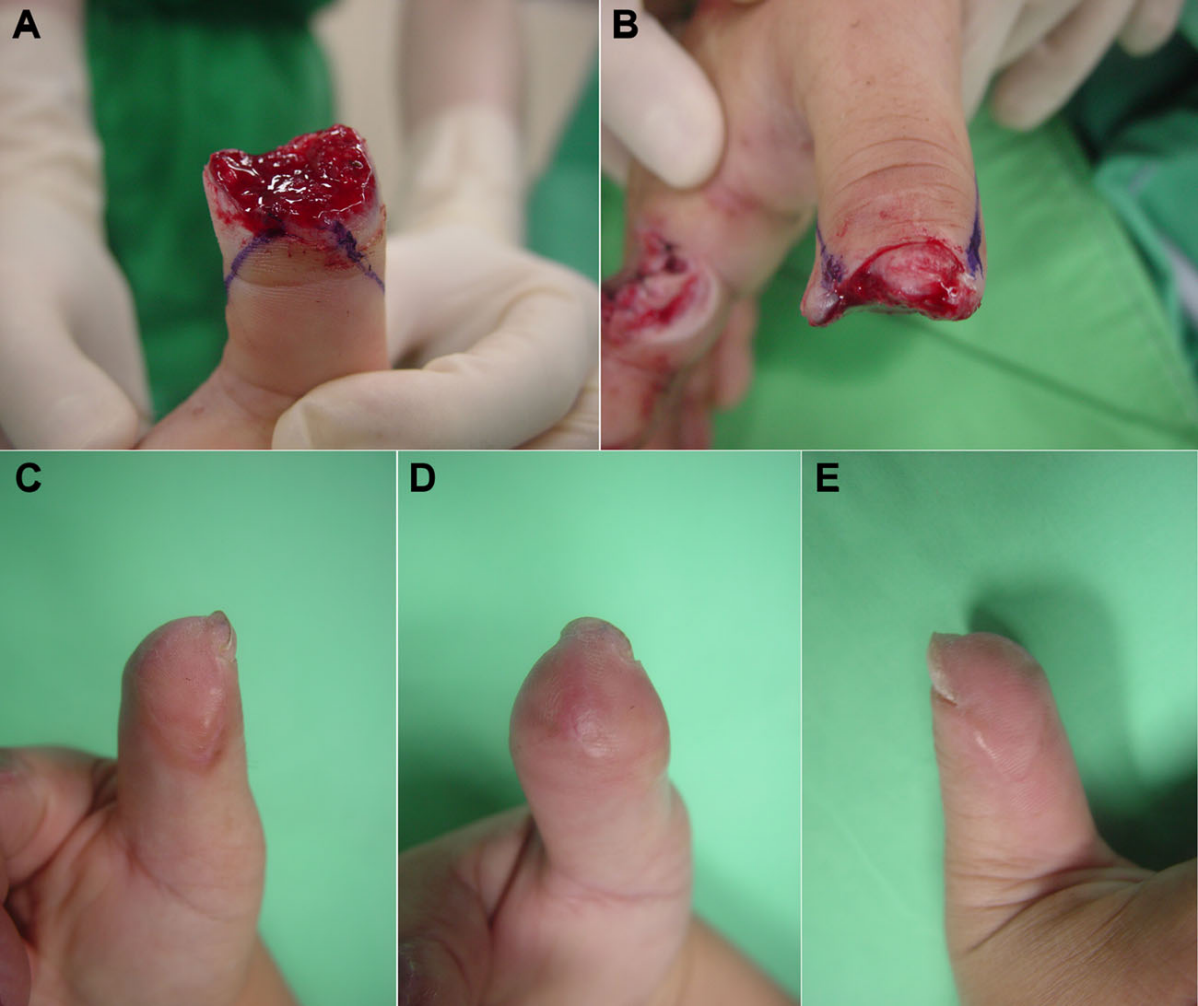
A local flap (lateral V‐Y advancement flap) reconstruction. (A), (B) A crush amputation injury with a distal right thumb loss and distal phalangeal bone exposure. (C), (D), (E) The bone‐exposed wound was reconstructed with a bilateral V‐Y advancement flap. The functional recovery and cosmetic result were good.

If bone is exposed in a distal digit injury, bone shortening and primary closure will quickly lead to wound healing, but this means that the digit will be shortened. A local or regional flap is suitable for salvaging the length of the digit for cosmetic and functional reasons. Several modifications of flap repair have been used in different clinical scenarios. A local flap for fingertip repair includes volar V‐Y advancement, lateral V‐Y advancement, or volar neurovascular advancement. The flap choice depends upon the angle of the fingertip injury and the individual surgeon's experience. For example, a volar V‐Y advancement flap is suitable for a dorsal oblique amputation, and a lateral V‐Y advancement flap can be used for a transverse amputation (Fig. 3).

**Figure 4:**
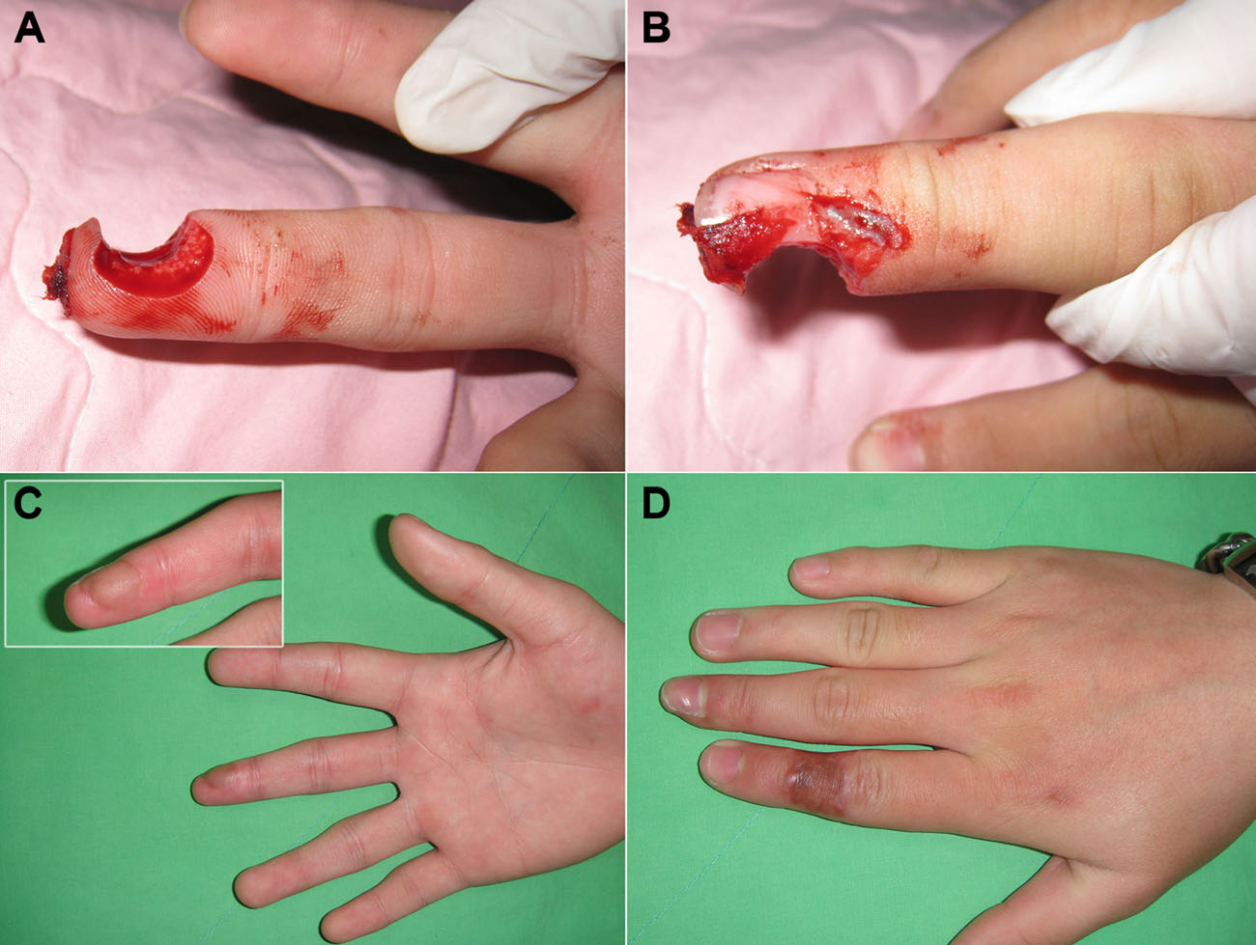
A cross‐finger flap reconstruction. (A), (B) A punch‐press amputation injury with a through‐and‐through defect over the right long‐finger pulp. The patient also had partial nailbed and nail‐plate loss with distal phalangeal bone exposure. (C) The wound with the exposed bone was reconstructed with a cross‐finger flap from the dorsum of the adjacent digit (index finger). The contouring of the reconstructed finger was good. (D) A full‐thickness skin graft from the patient's groin region covered the donor‐site wound of the index finger. There was almost no donor‐site morbidity.

**Figure 5:**
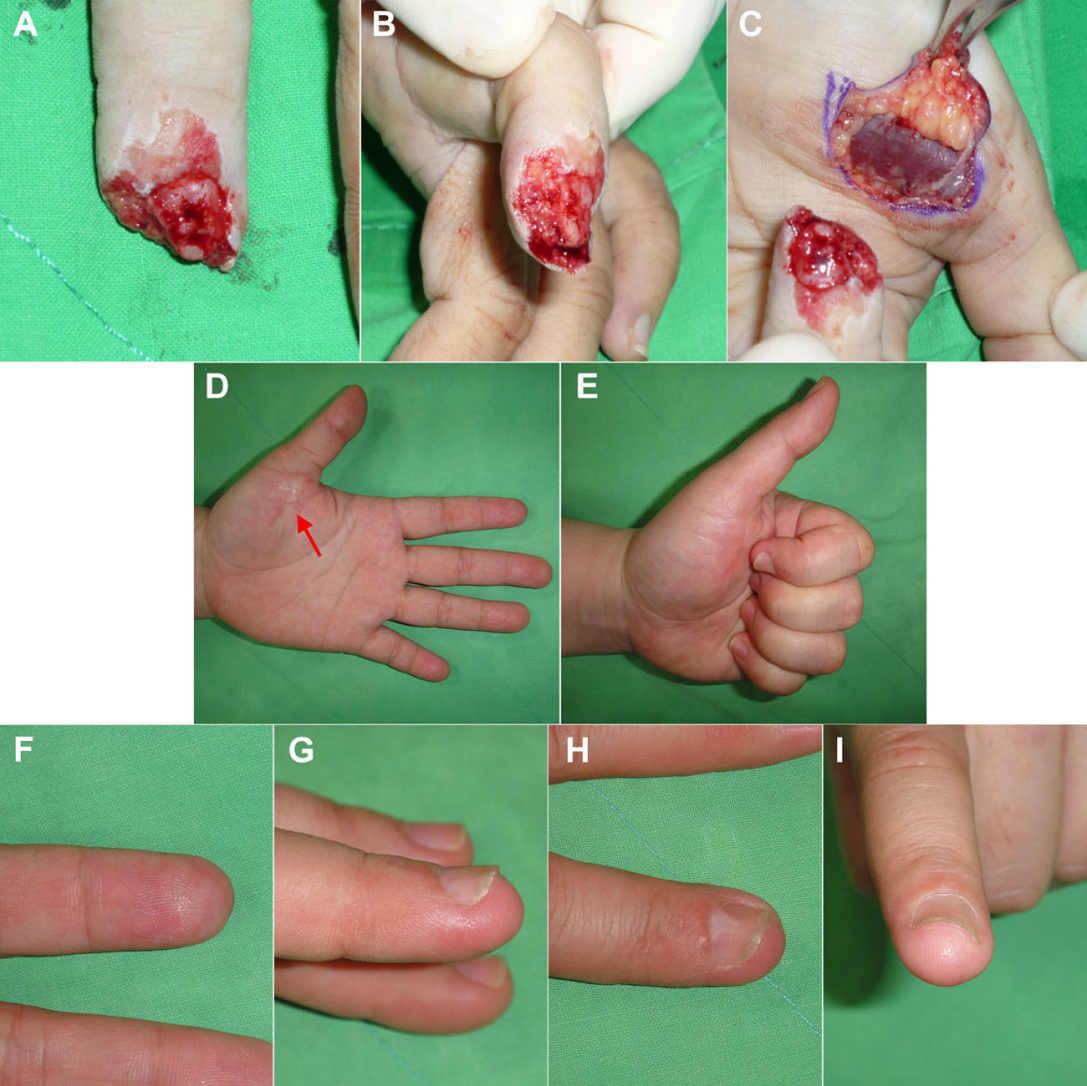
A thenar flap reconstruction. (A), (B) A crush amputation injury over the left index finger. Almost half of the distal phalangeal was lost, and the bone was exposed. (C) Using the “replace like with like” concept, a thenar flap was designed to reconstruct the amputated stump of the left index finger. (D), (E) The functional recovery was good, and there was only a very fine scar over the thenar donor site (arrow). (F), (G), (H), (I) The cosmetic results were excellent with similar volar glabrous skin texture.

**Figure 6:**
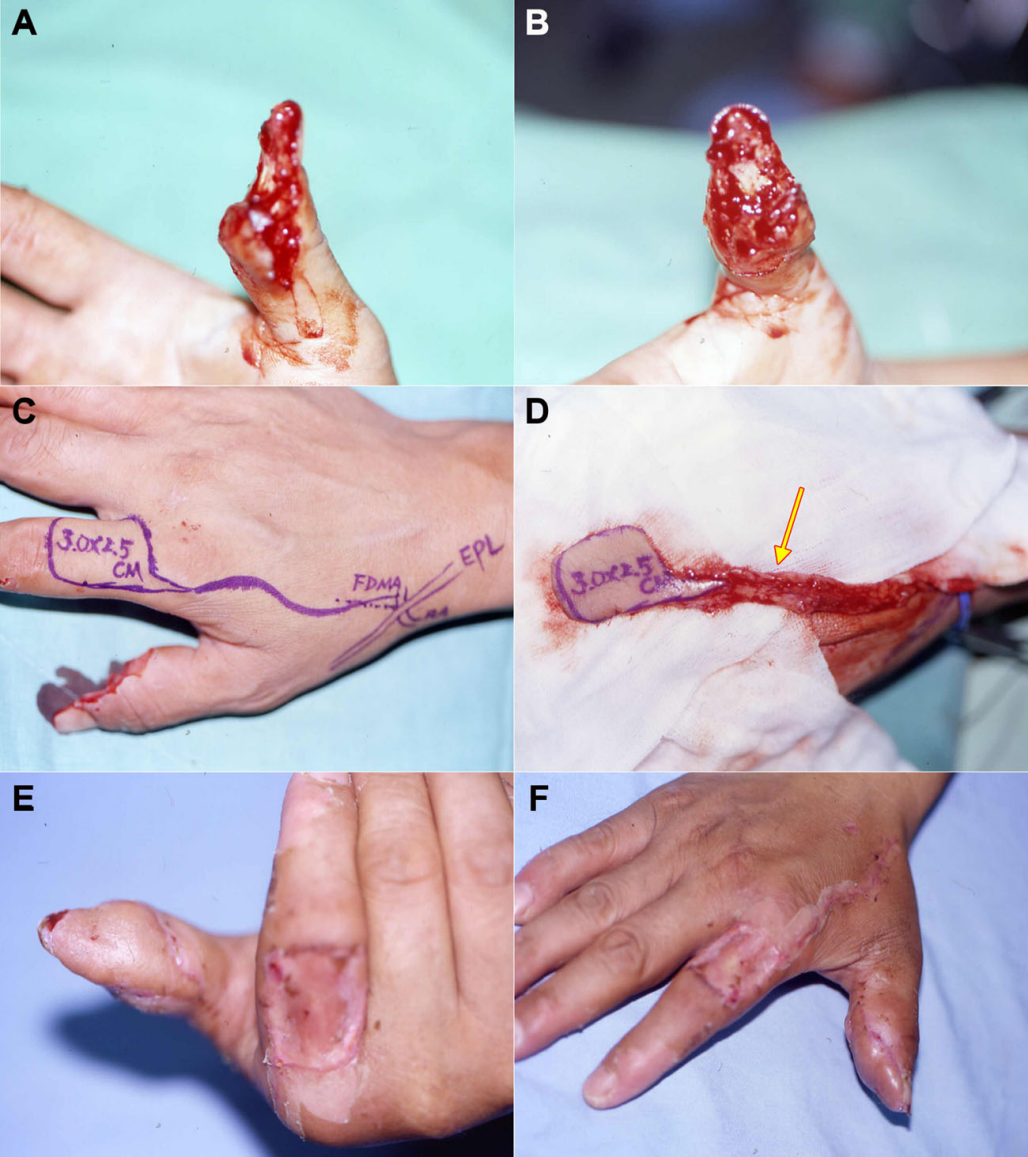
An innervated first dorsal metacarpal artery (FDMA) island‐flap reconstruction. (A), (B) A crush amputation of the total right thumb pulp with distal phalangeal bone exposure. (C), (D) An FDMA island flap with a sensory branch of superficial radial nerve (arrow) was elevated from the right index finger dorsum to reconstruct the amputated stump of the right thumb. (E), (F) The functional recovery and cosmetic results over the right thumb were good. The FDMA flap donor site was covered with a full‐thickness skin graft from the patient's groin region; it healed well.

If the fingertip injury is more extensive and local tissue is insufficient to cover a defect with exposed bone, regional flaps can be considered. The commonly used regional flaps to reconstruct fingertip tissue loss include the cross finger flap, thenar flap, and neurovascular island flap. The cross finger flap is indicated for a volar defect distal to a proximal interphalangeal joint (Fig. 4). With the modern reconstructive concept of “replace like with like,” thenar flap repair is a feasible choice for a distal digit amputation because it provides similar glabrous volar skin (Fig. 5). A neurovascular island flap, such as an innervated first dorsal metacarpal artery flap for thumb reconstruction (Fig. 6), may be considered if sensibility is the critical concern.

**Figure 7:**
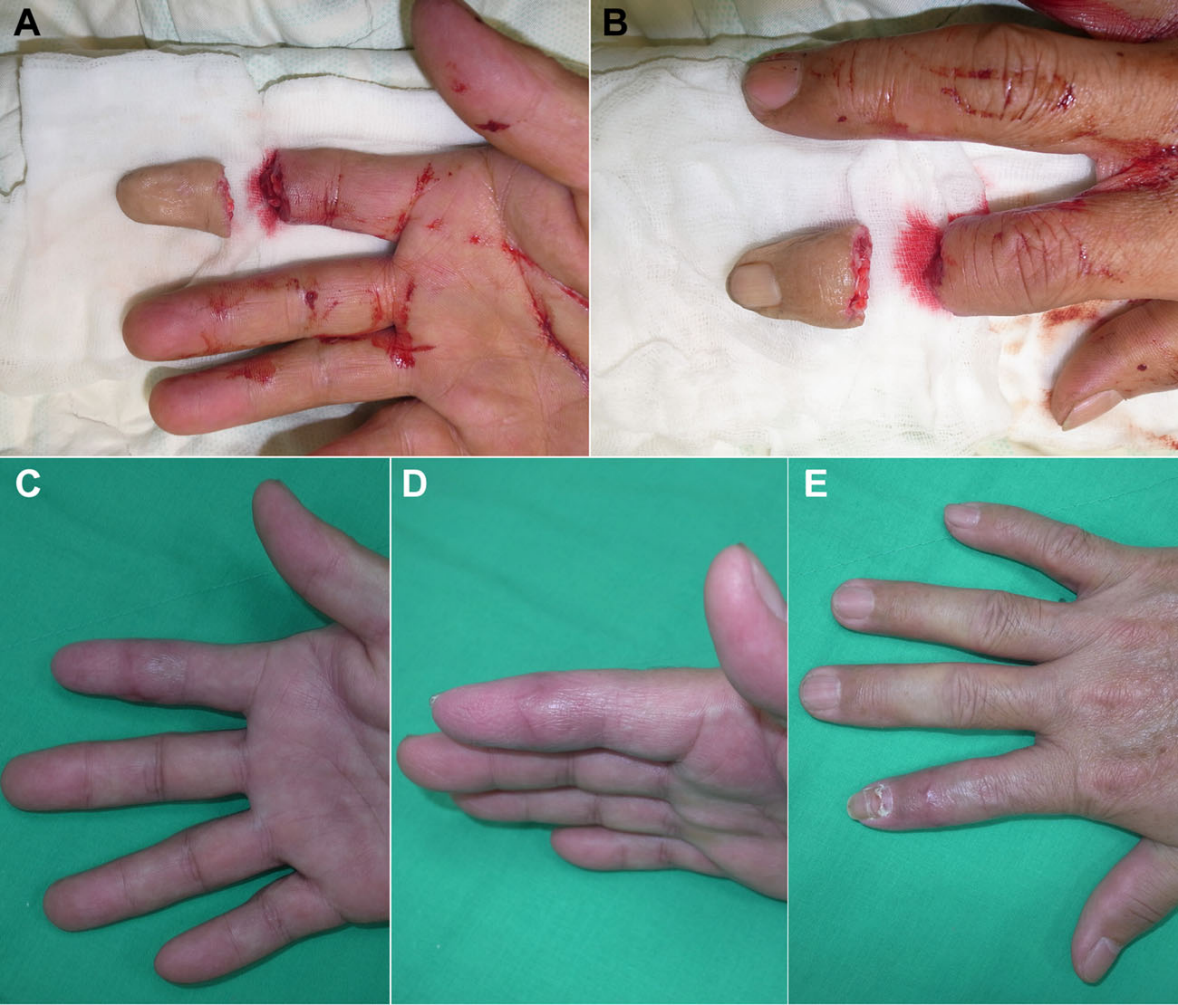
A replantation (single digit, crush type). (A), (B) A total crush amputation injury over the right index finger at the distal interphalangeal joint level. (C), (D), (E) The functional recovery was excellent after microsurgical replantation surgery.

**Figure 8:**
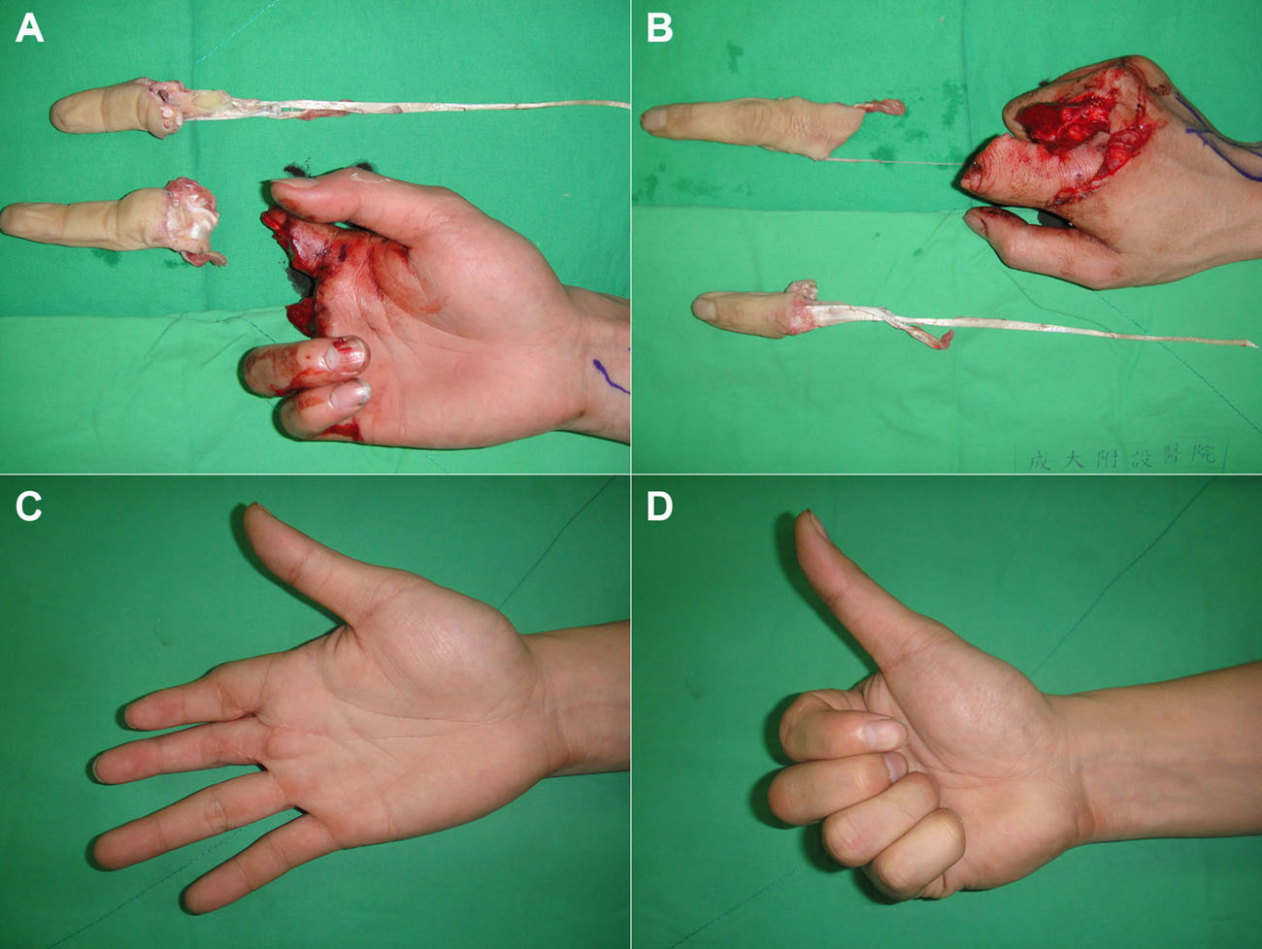
A replantation (multiple digits, avulsion type). (A), (B) A total avulsion amputation injury over the right index and long finger at the proximal interphalangeal level near the metacarpophalangeal joint. The index finger was avulsion‐amputated from the musculotendinous junction. (C), (D) The functional recovery and cosmetic results were excellent after microsurgical replantation surgery.

**Figure 9:**
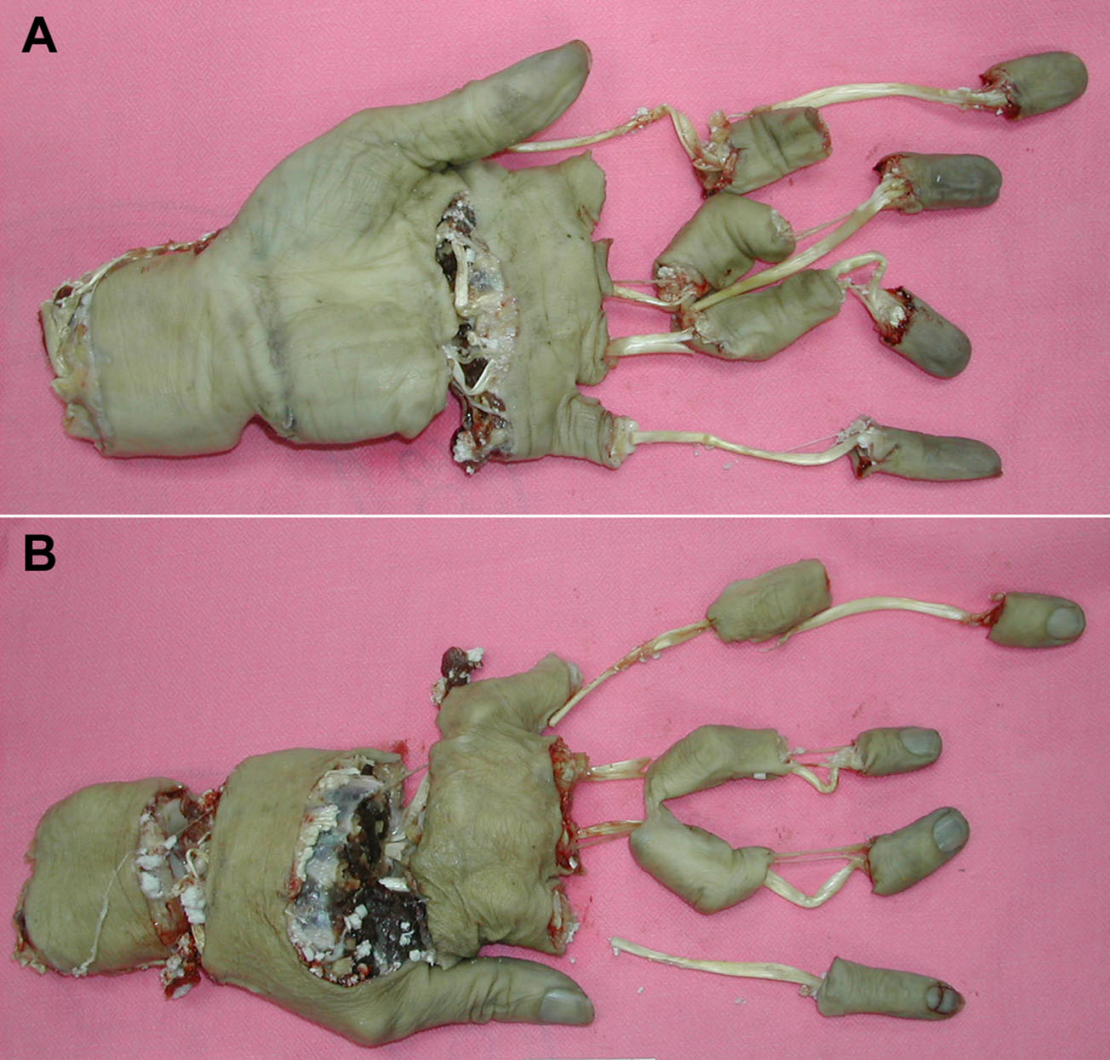
Unreplantable amputated limb. (A) A volar view of a multilevel avulsion amputation of the left forearm and hand. (B) A dorsal view.

For a more proximal digital amputation, although shortening the amputated stump and primary wound repair will provide quick wound healing, it causes significant deformity and functional loss of the hand. Patients always undergo severe physical and psychological trauma after an amputation. Therefore, plastic surgeons try to replant the amputated digits or limbs whenever possible (Figs 7, 8). Toe‐to‐thumb transplantation provides a feasible alternative and promising results if the amputated parts are unavailable or unsalvageable. In the Department of Plastic and Reconstructive Surgery at National Cheng Kung University Hospital, we have replanted 868 digits in 610 patients in the past 25 years (from July 1988 to December 2013). Seven hundred and sixty of those digits survived (88% success rate), and more than 80% of the replanted digits yielded good to excellent functional results, including a return to work rate of about 75% (Shieh et al. 1995, 1998, 2007, 2011; Yu et al. 2003; Lee & Shieh 2008; Lee et al. 2010, 2011). Nevertheless, not all digits or limbs will be replantable; in particular, those that are severely crushed and amputated (Fig. 9), those that underwent prolonged ischemia time, and those that were life‐threatening injuries will not. Regeneration or repair of human digits and limbs in the laboratory will undoubtedly generate hope for an alternative method, for example developmental biology or tissue engineering.

## Developmental Biology Approach

### General blastema concept

Why many non‐mammalian vertebrates can regenerate their injured and amputated body structures has been discussed for centuries. The mouse is an example of a mammal that has a relatively limited ability to regenerate its damaged or amputated digits.

How to make and form a blastema, an aggregation of progenitors for the new digit or limb, is the important lesson from amphibian regeneration, which has been studied with great interest (Muneoka et al. 2008). A blastema is a mass of multipotent progenitor cells that forms at a wound site in animals capable of regeneration. The missing part is regenerated from the blastema with mostly correct pattern and restored function. They are heterogeneous and inherit specific properties of the blastema cells from which they were derived; they are lineage restricted and, unlike pluripotent embryonic stem cells, have relatively limited differentiating potential (Kragl et al. 2009). However, it has been suggested that there are mechanisms that permit blastema cells to “transdifferentiate” into other cell types (Anderson et al. 2001).

Furthermore, blastemas have a positional memory that allows them to accurately restore the morphology of regenerated digits and limbs (Tamura et al. 2010). Individual regeneration models of salamanders, lizards, and mice are examined for regenerative cues for humans.

### Salamander limb regeneration

In 1768, the Italian physiologist Lazzaro Spallanzani first observed and uncovered the “magic” of salamander tail regeneration (Capanna 1999). Limb regeneration is an intrinsic trait of the urodele amphibians: salamanders, newts, and axolotls. They have the ability to regenerate amputated digits and limbs, regardless of the level of injury, throughout their whole life cycle (Han et al. 2005). Salamander limb regeneration involves the following steps (Fior 2014). After an amputation has occurred, epithelial cells migrate to cover the stump. They then thicken and form an apical epidermal cap (AEC). Under the AEC, a cluster of undifferentiated cells aggregate to form a blastema, which is required for regeneration (Gardiner et al. 1986). Recent research (Godwin et al. 2013) reports that macrophages are necessary for salamander limb regeneration. Macrophage deprivation immediately after an amputation leads to collagen deposition that will form fibrous stumps rather than limb buds.

The newt anterior gradient protein, which is a secreted ligand for Prod1, is expressed by the Schwann cells of injured axons during regeneration and mediates blastemal cell proliferation. The blastemal cell expresses the surface protein Prod1 for proximodistal positional identification and signal transduction to regulate the process (da Silva et al. 2002; Kumar et al. 2007).

### Lizard tail

Caudal autotomy or self‐amputation of the tail is a strategy that lizards use to escape from predators. However, to lizards, tails are important for locomotion, sexual interaction, and psychosocial status (Clause & Capaldi 2006).

Once autotomy or even surgical amputation occurs, blood clot formation and skin contraction shrink the wound on the fracture plane which later leads to re‐epithelialization with adjacent epidermal cells growing across the wound surface. Beneath the neo‐epidermal is then the aggregation of a mass of proliferating cells, the so‐called blastema. The blastema cells extend distally and laterally to cover previous tissue loss area (McLean & Vickaryous 2011; Delorme et al. 2012). The ependymal tube of original spinal cord elongates within the blastema and acts as guidance for lizard tail regeneration (Alibardi & Miolo 1990).

While sources of blastema cells in lizard tail regeneration models are still unclear, a reasonable deduction is that the contribution cells are also a collection of heterogeneous lineage‐restricted cells just as in other regeneration‐competent vertebrates (McLean & Vickaryous 2011; Delorme et al. 2012).

Caudal regeneration in lizards results in a morphologically and superficially nearly identical replica: the newly formed but imperfect tail has the demerit of having incompletely restored the spinal cord with unsegmented hollow cartilaginous vertebrae and a notochord. Even so, tail regeneration is superior to limb regeneration in lizards, which induces “a massive inflammatory response in the limb” and subsequent severe scarring (Alibardi 2010).

### Mouse digit regeneration

Zebrafish can fully regenerate many types of tissue. Salamanders regrow injured limbs with restored and intact functions. Reptiles have the ability to rebuild morphologically similar but only mildly functional imperfect tails. However, in more highly evolved vertebrates, mammals, for example humans and mice, have relatively limited regeneration repertoires restricted to digit tips (Al‐Qattan et al. 2014).

When a neonatal mouse sustains an amputation, it forms a wound epithelium. In adult mice, however, osteoclasts first degrade the bony stump, and then epidermal cells migrate to close the wound (Fernando et al. 2011). Next, both in neonatal and in adult mice, some proliferated cells aggregate to form a blastema beneath the wound epithelium. Finally, regeneration ends with the redifferentiation of the distal digit tissue (Han et al. 2008; Fernando et al. 2011).

Most of the blastema cells involved in mouse digit regeneration are derived from tissue‐resident stem cells and are thought to be a collection of multiple lineage‐restricted cell populations (Lehoczky et al. 2011; Rinkevich et al. 2011). Moreover, in both human fetal (Allan et al. 2006) and mouse neonatal (Han et al. 2003) digit tip amputation, the remaining nail bed produces Msx1, a transcription factor that regulates downstream bone morphogenetic protein 4 expression, which is vital for proliferation and dedifferentiation in vertebrate appendage regeneration.

Successful digit regeneration is level‐dependent; that is, an amputated terminal digit can regenerate into an acceptable replacement, but an amputation more proximal to the distal third phalanx will end with scar formation (Neufeld & Zhao 1995; Reginelli et al. 1995).

## Challenges of the Developmental Approach for Digit and Limb Regeneration

Animal‐model regeneration study findings concur that, to maintain a highly variegated potential of regeneration, a species must pay the price of being evolutionarily relatively primitive. The more complex the species, the more regulatory pathways there are to control wound healing, blastema formation, and cell differentiation and dedifferentiation (Sánchez Alvarado & Tsonis 2006).

Gene regulation has been a major focus of the developmental approach, but the notion has turned out to be an oversimplification. The most impressive regeneration model belongs to the salamander, but its genome has not been sequenced. Identifying the specific genes that regulate the specific functions will help us better understand the developmental aspects of digit and limb regeneration in humans. Some researchers postulate that the wound healing process may be a determinant of regeneration. Epidermal cells migrate to cover the amputated surface and form AECs within 24 h in salamanders, but it takes much longer in mice. Delayed AEC formation may reduce regeneration ability (Al‐Qattan et al. 2014). In addition, excessive scarring hinders regeneration. Therefore, transforming growth factor β expression in collagen formation is suppressed during limb regeneration (Lévesque et al. 2007).

Regeneration shares many mechanisms with embryonic development. Numerous genes that regulate regeneration pathways were originally set up for embryonic limb development. Therefore, some digit‐ and limb‐generative genes are switched off after embryogenesis and never turn on again. These are the genes that turn the limb bud into a morphologically and functionally perfect human arm at the molecular, tissue, and organ levels, for example in muscular agonist−antagonist pair establishment, and for axial rotation to produce an opposable thumb (Carter & Wong 1988; Hughes & Salinas 1999). How to control these now non‐functioning regenerative steps is an arduous challenge.

Immune signaling pathways may affect the regenerative and repair process, for which macrophages are crucial. There are various functional phenotypes in response to tissue injury and repair. M1 phenotype macrophages respond to inflammatory and antimicrobial events, and M2 phenotype macrophages potently have an important anti‐inflammatory role in wound healing (Mosser & Edwards 2008; Murray & Wynn 2011; Novak & Koh 2013). Macrophage infiltration promotes blastema formation and limb outgrowth in salamanders, but it leads to scar formation in mammals. Although macrophages in both species produce similar cytokines and chemokines, salamander macrophages induce inflammatory and anti‐inflammatory processes simultaneously, but mammalian macrophages induce anti‐inflammatory reaction at later wound healing processes (Godwin et al. 2013). It might be possible to artificially regulate this immune‐cell signaling in mammals by directing macrophages to the M2 subset.

The difference in scale between generating embryonic limb buds and regenerating damaged and amputated adult limbs is tremendous. It seems inappropriate to create a tiny limb on an adult amputated stump surface. Brockes and Kumar (2005) reported that specific underlying mechanisms were required for newts to regenerate size‐compatible limbs. However, it takes the human body about 15 years to create an adult arm. How to manipulate and artificially accelerate the development and growth of adult digits and limbs is still the stuff of science fiction.

## Tissue Engineering Approach

### General tissue engineering concept

Tissue engineering is a late 1980s multidisciplinary merger of biology, materials science, and engineering (Langer & Vacanti 1993; Kaihara & Vacanti 1999; Lalan et al. 2001; Shieh & Vacanti 2005). It aims to deal with the shortage of organ resources for transplantation, to reconstruct injured appendages, and to manage congenital anomalies. Faced with the problems of tissue loss, numerous modalities have been proposed as tissue substitutes. The two most common options are alloplastic prosthesis implantation and autologous grafts, each of which has pros and cons (Shieh et al. 2004). Tissue engineering offers many pros because it has the potential to overcome the disadvantages of current treatment plans and to establish a new functional structure (Salgado et al. 2004). The general strategies of tissue engineering are (1) to isolate the designated cells based on replicated tissue; (2) to anchor those cells to a suitable scaffold; and (3) in an appropriate environment to immerse the cells with appropriate bioactivators (e.g., growth factors) (Langer & Vacanti 1993). Cells used in tissue engineering can be isolated from autologous, allogenetic, or xenogenetic resources, either from donor tissue cells or progenitor and stem cells. Ideal cell sources should be easily accessible and reproducible (Shieh & Vacanti 2005). A scaffold is a three‐dimensional and highly porous biomaterial structure that allows the cells to function in a native environment for cell attachment, migration, and loading, or it is a structure that contains the bioactivators. The scaffold's porosity permits adequate nutrition and oxygenation diffusion. The scaffold also must be biodegradable but without losing resistance to mechanical stresses (Hutmacher 2000; Berthiaume et al. 2011).

Applications of tissue engineering for digit and limb regeneration in humans involve replacing various structures: skin, cartilage, bone, vessel, nerve, tendon and ligament, and muscle. The next seven subsections discuss individual tissue engineering work related to digit and limb regeneration and point out current critical obstacles for combining these complex structures.

### Skin

The skin is an essential barrier that protects the body from exogenous assaults and prevents water and solutes from evaporating (Proksch et al. 2008). Therefore, how to deal with skin loss and skin defects is often a serious problem. The healing process for skin wounds larger than 1 cm and as deep as the dermis may be impaired and thus require therapeutic intervention (Berthiaume et al. 2011). Skin grafts have been one successful clinical management strategy. There are many limitations in skin‐graft technology, however, and tissue engineering is a relatively mature alternative compared with other methods. Skin substitutes are used to accelerate the normal wound healing process but without the disadvantages of skin grafts, for example the donor site availabilities in autogenic grafts or immune rejection in allogeneic grafts (Lee 2000). Shakespeare (2005) identified and summarized the functions of tissue‐engineered skin into (1) protection: forming an impermeable barrier to hinder the invasion of microorganisms and the evaporation of body fluids; (2) procrastination: covering the wound initially to delay the wound‐closure operation; (3) promotion: delivering dermal matrix, cytokines, and growth factors to the wound bed to accelerate wound healing; and (4) provision: incorporating new substances, such as collagen and cultured cells that accelerate tissue repair and persist during wound healing.

The first cultured skin can be traced back to 1975, when Rheinwald and Green (1975) reported that they had cultured human keratinocytes in murine fibroblasts in vitro. Many commercialized bioengineered skin substitutes are available now, and individual products vary based on the source of the cells, method of delivery, and supplementary substrates such as fibroblasts and matrix proteins. Advances in stem cell biology, genome editing skills, and grafting techniques are improving genetically engineered skin grafts (Sun et al. 2014).

However, there are still no permanent skin substitutes. All skin defects require final repair (Shevchenko et al. 2010). Although the artificial skin accelerates the natural process of wound healing, the long‐term stabilities, wound healing, and aging process of artificial skin may be different from those of natural skin. Furthermore, the possibility of carcinogenesis is also a concern because tissue‐engineered keratinocytes are usually activated and “hyperproliferating” (O'Leary et al. 2002; Alrubaiy & Al‐Rubaiy 2009).

### Cartilage

Joint cartilage reduces joint friction and is a natural shock absorber. Injuring this cartilage may lead to joint pain and functional impairment (Little et al. 2011); thus, a demand for tissue‐engineered cartilage replacements soon developed. Therapeutic applications for other types of cartilage include reconstruction of congenital auricular anomalies, the nasal alar, and the temporomandibular joint disk, and replacement of the trachea. Cartilage is an avascular tissue with a low metabolic demand; it relies on diffusion to obtain nutrients. Because of the unique characteristics of cartilage, using tissue engineering to repair and regenerate cartilage is probably less problematic than using it for repairing and regenerating other types of tissue: the vascularization of the target structures is less critical. Moreover, the lower metabolic requirements of cartilage mean that we need not be constrained by concerns about diffusion (Lalan et al. 2001). The first autologous cultured‐chondrocyte transplantation (Brittberg et al. 1994) was done more than two decades ago. The chondrocytes were subsequently produced and sold commercially as Carticel (Genzyme Biosurgery, Cambridge, MA, USA). This modality requires extracting chondrocytes from uninjured cartilage and then implanting them into the affected joint. This epoch‐making technology using chondrocyte transplantation has been used to make functional improvements in full‐thickness defects in the articular cartilage of human knee joints. Vacanti et al. (1992) described how to use an ear‐shaped scaffold to grow new cartilage in a defining shape of a human ear. However, the defining shape either shrank or was distorted in up to 40% of the reported tissue‐engineered constructs. This led to a search for suitable and reliable tissue sources and biomaterials to improve the integrity of the constructs. We previously reported (Shieh et al. 2004) promising in vitro and in vivo neocartilage formation techniques for auricular scaffold fabrication in a nude mouse model, and first reported on different biodegradable biomaterials and the longest (10 months) in vivo trials for auricular tissue engineering. We found, however, that the tissue‐engineered cartilage was severely deformed in an immunocompromised xenograft and autologous immunocompetent rabbit model; histological examination showed that inflammatory cells had infiltrated the constructs and compromised their integrity. Numerous challenges remain. Current problems include ensuring that implants are securely fixed to the underlying tissue; that the dedifferentiation rate of the cultured cells, which may affect the type II collagen density, is acceptable; and that the matrix is appropriately stratified by maintaining sufficient cushions to avoid injuries from mechanical stresses (Berthiaume et al. 2011). Moreover, additional studies are required on the biochemical, immunological, and biomaterial interaction between the host and the implants (Bichara et al. 2012).

### Bone

Osseous tissue has good regenerative ability. However, when facing large bone defects caused by a tumor resection or a nonunion fracture, how to reconstruct those defects may be troublesome. The current gold standard is an autologous bone graft, which can integrate well into a bony host structure without undergoing immune reactions or transmitting diseases from the allograft or xenograft (Burg et al. 2000; Salgado et al. 2004; Stevens 2008). Because of limited sources of autologous bony tissue and allogeneic donor morbidities, bone tissue engineering was also developed in the 1980s. The source is mesenchymal stem cells (MSCs) isolated from bone marrow (Friedenstein et al. 1987). Unlike cartilage, bone tissue has a higher metabolic rate; therefore, a vascular supply is vital for bone tissue engineering. Nevertheless, inducing endothelial cells to perch on a scaffold and then to develop blood vessels is still in the animal model stage of development. Although numerous bone tissue engineering experiments have succeeded with small rodents, the success rate has not been matched in experiments on large animals with segmental bony defects (Stevens 2008).

Bruder et al. (1998) found that rapid bone regrowth occurred in the interface between the host bone and the implant (a porous ceramic scaffold seeded with MSCs in adult dogs), and similar success was reported in sheep models (Kon et al. 2000; Petite et al. 2000). When attempting to use this with humans in clinical settings, however, there are many problems. The first reported clinical use (Quarto et al. 2001) occurred at the beginning of this century. It was established by placing ex vivo culture‐expanded osteoprogenitor cells isolated from bone marrow on macroporous hydroxyapatite scaffolds, and then implanting them into the large bony defects of three patients. All had good outcomes, but there are numerous problems that need to be solved before the method can become a standard strategy: vascularization, homogeneous distribution of nutrition and oxygen, the release kinetics of growth factors, and the intercellular and intracellular signaling pathways of bone regeneration (Burg et al. 2000; Salgado et al. 2004; Cancedda et al. 2007; Stevens 2008).

### Blood vessels

Synthetic nondegradable materials for bypass surgery to rescue patients from complex cardiovascular diseases have been used for years, but only in vessels larger than 6 mm in diameter because of the acute thrombogenicity of the graft, fibrous intimal hyperplasia in the anastomosis site, easy aneurysm formation, and progression of atherosclerosis when used for low‐flow, small‐caliber vessel grafts (Conte 1998; Klinkert et al. 2004). In addition, these materials have no growth potential, which limits their use with children (Atala 2005). Tissue engineering blood vessels in vitro were introduced by Weinberg and Bell (1986). The first blood vessel construct was manufactured using polyethylene terephthalate (Dacron) as scaffold material to create stratified collagen that was seeded with endothelial cells and smooth muscle cells. Degradable scaffolds with autologous cells were soon being used in experiments on dogs and sheep (Shinoka et al. 1995; Watanabe et al. 2001).

A tissue‐engineered blood vessel graft using a degradable scaffold was first used clinically on a 4‐year‐old girl with pulmonary atresia and a single right ventricle to reconstruct her occluded pulmonary artery. This conduit yielded a good result with no evidence of occlusion or aneurysm formation in the following 7 years (Shin'oka et al. 2001). Nonetheless, tissue‐engineered vessels must still be used only on large‐caliber (>6 mm) vessels.

### Nerve

Neural tissue repair and regeneration for salvaging peripheral nerve injuries is a currently attractive topic. The delicate alignment of nerve ends with a good blood supply and soft tissue coverage is critical for optimizing nerve recovery, and gaps greater than 2.5 cm will usually need nerve grafting (Griffin et al. 2013). Although conventional autologous neural grafts are still standard therapy, novel tissue engineering techniques have emerged to overcome the demerits of nerve autografts: the shortage in the availability of grafts, neural‐size mismatches between donors and recipients, aberrant regeneration, and donor‐site functional morbidities. Artificial nerve conduits have therefore appeared as an alternative for repairing peripheral nerve defects shorter than 3 cm (Pabari et al. 2014). The host−graft immune rejection of allogeneic nerve grafts from cadavers is another concern. Acellular grafts have been developed, but these delay nerve regeneration and yield a low degree of functional recovery. Neural tissue engineering aims at providing a regeneration‐friendly environment by introducing three‐dimensional biosynthetic scaffolds, which are extracellular matrix analogues. An ideal scaffold is a guide and provides geometric, electrical, and chemical signals to regulate the migration and adhesion of neural cells. The characteristics of the synthetic scaffold and the concentration of the growth factors have a tremendous influence on neural stem cell differentiation (Nakajima et al. 2007). Therefore, the concentration of growth‐factor delivery and the retention and sustained release of these factors in the injury site is an important and difficult problem (Levenberg et al. 2003). Although some growth‐factor carriers have been manufactured, acid degradation that possibly causes protein inactivation is the major concern (Xu et al. 2002). Other problems that have hampered the development of clinical applications for neural tissue engineering include the long‐term safety of the biomaterial and how to combine all approaches to promote nerve tissue regeneration (Subramanian et al. 2009).

### Tendons and ligaments

Tendons and ligaments are dense, fibrous connective tissue, and attach bone to muscle and bone to bone, respectively. Their main function is to stabilize joints and improve locomotion. Exercise‐ or degenerative‐disease‐induced tendon and ligament damage is common and currently increasing (Khan et al. 2014). However, the standard therapeutic strategies of conservative corticosteroid injections and surgical intervention are unable to recover the natural function of tendons and ligaments; “repaired” tissue tends to have inferior mechanical and biochemical properties. Surgical treatment replaces damaged tendons and ligaments with autografts, allografts, xenografts, or prosthetic devices. In addition to unsatisfactory functional outcomes, there are concerns about donor‐site morbidities, immune rejection, and infection risks with tendon allografts and xenografts. The ultimate goal of tendon‐ and ligament‐tissue engineering is full restoration (Yang et al. 2013).

The tendon fibroblast, the tenocyte, is the priority cell source of tendon‐ and ligament‐tissue engineering. However, harvesting autologous tenocytes may lead to secondary tendon injury and donor‐site morbidity (Yang et al. 2013). The dermal fibroblast, an alternative, is easier to harvest. Dermal‐fibroblast‐engineered tendons share almost identical morphologies and mechanisms with tenocyte‐engineered tendons (Van Eijk et al. 2004). Another option is the adult MSC, which produces an engineered tendon with better tensile strength and more collagen production (Yang et al. 2013). In addition to harvesting cells, a suitable scaffold for cell adhesion and migration with accurate biomolecular signals and either static or cyclical mechanical stimulation is necessary for successful tendon engineering (Van Eijk et al. 2004).

Despite the work already done in this field, synthetic tendons and ligaments are still not a first‐line therapeutic option because of their discrepant mechanical properties and difficult host‐tissue incorporation (See et al. 2013).

### Skeletal muscles

Skeletal muscle cell is a multinucleated syncytium, and is formed by the fusion of striated myotubes. Its main function is to execute voluntary movement and maintain the structural contour of the body. Once skeletal muscle is injured, stellate cells, located beneath the basal lamina, repair and replace the damaged muscle cells (Yan et al. 2007). Skeletal muscle may be lost because of a traumatic injury, tumor ablation, or myopathy‐induced functional impairment. One of the clinical modalities for treating major skeletal muscle damage is vascularized free functional muscle transplantation. However, this treatment may cause donor‐site morbidity, poor functional restoration, and insufficient tissue mass (Stern‐Straeter et al. 2007).

**Figure 10:**
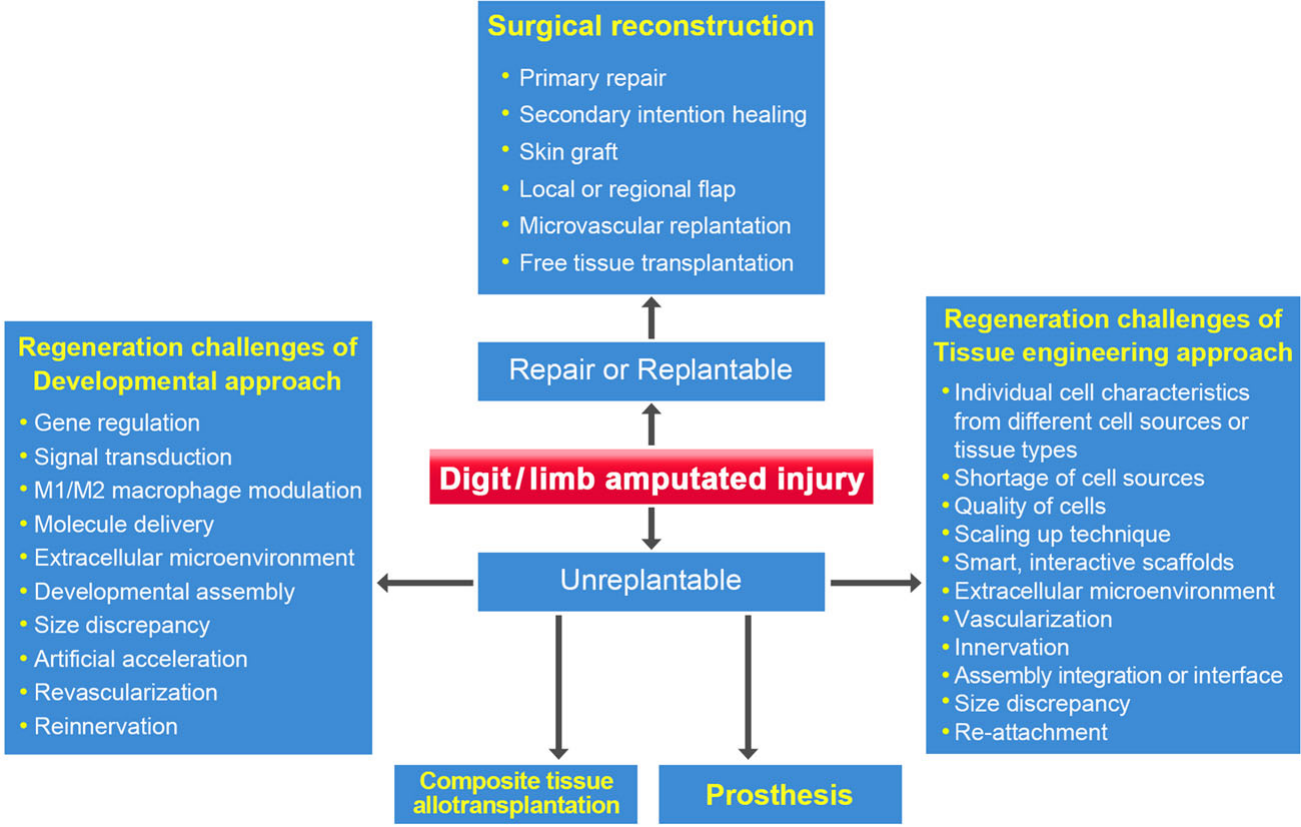
The strategies and challenges for the regeneration and repair of human digits and limbs.

**Figure 11:**
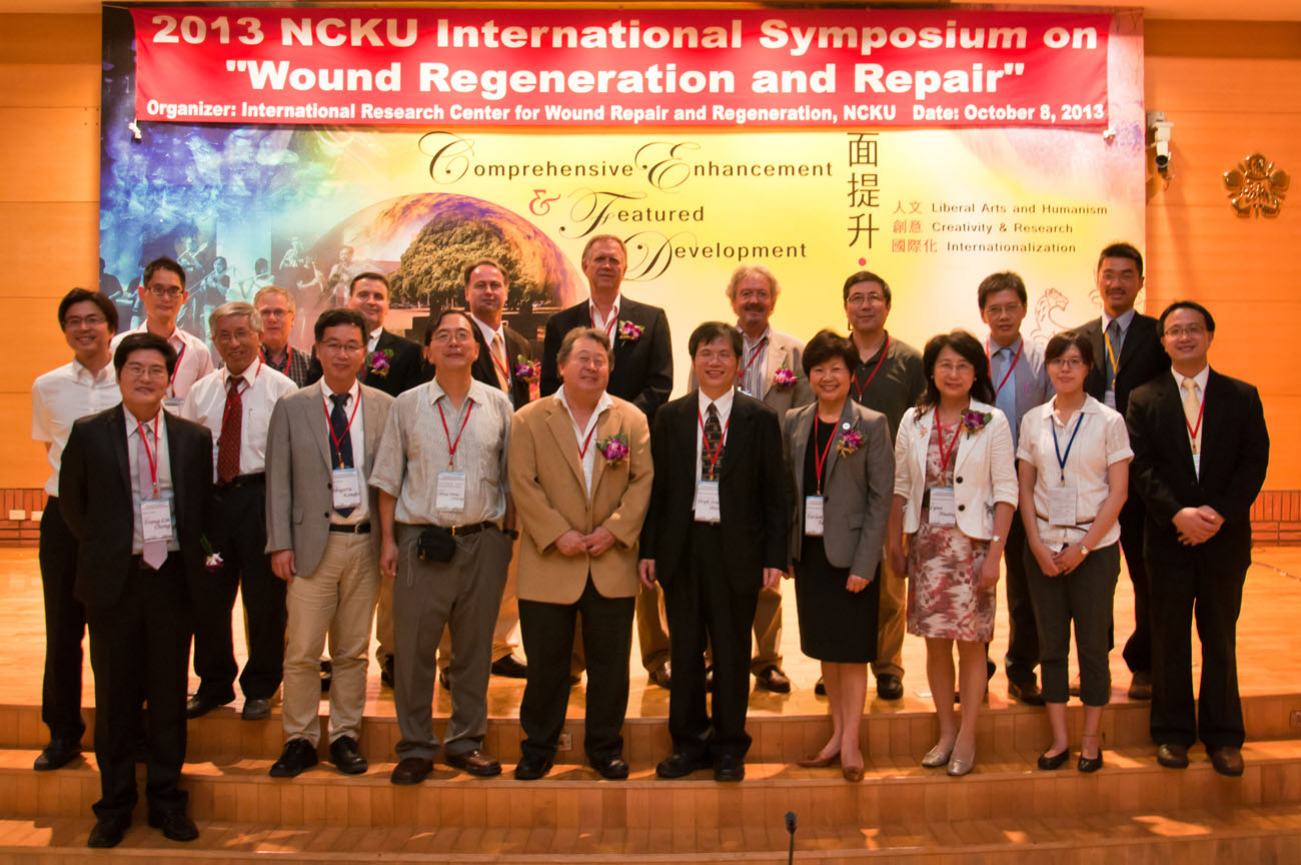
A group photograph of the International Symposium on Wound Regeneration and Repair held on 8 October 2013 at National Cheng Kung University, Tainan, Taiwan. The symposium was organized by the International Research Center for Wound Repair and Regeneration (iWRR) at National Cheng Kung University. Many internationally renowned professors in the field of regenerative medicine attended the meeting. The discussion on digit and limb regeneration was heated and exciting.

Skeletal muscle tissue engineering is an attractive alternative to autologous muscle transplantation. Satellite cells, which are easily obtainable from muscle biopsies and have regenerative potential, are the primary source. Isolation and in vitro proliferation of satellite cells have been successfully achieved in animals and humans (Blau & Webster 1981). Based on previous experimental results, satellite cells have more outstanding myogenesis performance than immortal myogenic cell lines (Bach et al. 2003, 2006). Other cell sources include MSCs, hematopoietic stem cells, and embryonic stem cells (Dusterhoft & Pette 1990).

There are in vitro and in vivo approaches in skeletal muscle tissue engineering. The in vivo approach is cultivation and expansion of biopsied muscle cells in vitro and then reimplantation and differentiation in vivo. In contrast, the in vitro approach is aimed at a three‐dimensional construct, either using a three‐dimensional scaffold or by co‐cultivating fibroblasts and then synthesizing and expanding them under suitable in vitro conditions. Some biomolecules (e.g., insulin, insulin‐like growth factors, etc.) and mechanical or electrical stimuli are introduced to imitate the in vivo environment (Stern‐Straeter et al. 2007).

Skeletal muscle tissue has a capacious metabolic rate. Therefore, oxygen and nutrition diffusion capacities are an important concern for tissue mass expansion. Tanaka et al. (2000) constructed a vascularized‐tissue‐engineered skeletal muscle flap in rats using an arteriovenous shunt loop. Levenberg et al. (2005) also showed that prevascularization was effective for increasing cell survival rates in mice. Although skeletal muscle tissue engineering has improved in recent years, most of the method's hypotheses still require testing in animal models. Additional research is necessary to overcome technical obstacles.

## Challenges of the Tissue Engineering Approach for Digit and Limb Regeneration

Tissue engineering has been a productive field for several decades. At present, we can generate many types of tissue in culture, including all the components essential for an artificial extremity. However, regrowing a human digit or limb remains a dream. The gold standard for tissue repair is still autologous tissue. For example, despite the relative maturity of skin tissue engineering, skin substitutes are temporary. The cultured tissue is inferior to host‐regenerated tissue. Specifically, artificial skin has neither neurosensory functions nor standard dermal accessories like hair follicles, sweat glands, sebaceous glands, etc., and it is usually extremely fragile; it has been described as embryonic‐like rather than adult‐like (Ricci 2013).

Scaling up production is also an obsession with scientists nowadays. The shortage of cell resources is another hurdle. Most starter cells for tissue engineering are autologous lineage committed, like fibroblasts, keratinocytes, and chondrocytes. However, because of their insufficient proliferative capacity and difficult approachability, lineage‐committed cells are not always the optimal choice (Lewandowska‐Szumiel & Kalaszczynska 2013).

Surface area to volume must be matched for an effective mass transfer to insure the delivery of adequate oxygen and nutrients. Tissue mass measured beyond 2−3 mm^3^ is prone to necrosis because of insufficient nutrients, limited gas exchange, and poor elimination of waste metabolites (Langer & Vacanti 1993). To solve the problem of the finite distance of nutrient and oxygen diffusion, vascularization must be established and integrated into regenerated digits and limbs. Potential solutions currently being tested to overcome this intrinsic constraint are (1) adding angiogenesis factors, (2) introducing endothelial cells together with planned‐culture cell types, and (3) vascularizing the scaffold before implanting the cells (Shieh & Vacanti 2005). Decellularized but preserved vascular scaffolds from a rodent's live heart and lungs succeeded in restoring organ functions after the cells had been replenished (Ott et al. 2008, 2010). This alternative approach also showed that vascular structure is vital for organ regeneration and is still one of the major obstacles for artificial organ development.

Once we successfully combine all required tissue types for manufactured extremities, however, the size discrepancy between the host's remaining parts and the transplant structures may still exist. Reactivating switched‐off genes encoded for fetal regeneration is another impediment because, if the generated structures are fetal or embryonic sized and much weaker than adult structures, they will require about 15 years to become full sized. Scientists hope to resolve this problem by discovering or developing biologic agents to accelerate growth (Merolli 2013; Ricci 2013).

Another problem to solve is how to integrate multiple tissue types into anatomical and functional structures, and how to deal with the interface between different types of tissue. The final frontier is implanting the engineered structures in the host, which will require a difficult transplantation surgery with a sophisticated neurovascular anastomosis. Furthermore, for functional nerve regeneration, the cell bodies of motor nerves and dorsal root ganglions of spinal nerves that provide sensory functions are both far away from the transplanted structures. Regenerating nerves across long distances to the target tissue will undoubtedly be daunting for a few more decades (Ricci 2013).

Aforementioned complex issues, for instance, gene regulation and cell signaling of cell sources, engineered tissue−tissue interface, dimensional discrepancy, and anastomosis of engineered nerves and vessels with host neurovascular structures, etc., are all crucial factors affecting functional engraftments between engineered tissue and host tissue.

## Summary

Digit and limb injuries usually have profound effects on their victims. Not only do they suddenly have functional disabilities, but many are also pummeled by an understandable cosmetic distress. Surgical treatments have done the best to minimize the inconvenience caused by disability, but neither a perfect functional nor cosmetic recovery can always be expected.

Developmental biology studies have uncovered the secrets of digit and limb regeneration in animals relatively low on the evolutionary ladder (salamanders, newts, and axolotls), but we are not yet able to duplicate their primitive magic in the human body. Difficulties in gene regulation, molecule delivery, and growth acceleration pose currently insurmountable technical problems for regrowing human digits and limbs in the laboratory, problems that, optimistically, might be solved within a few decades or by the end of this century or, probably more realistically, will remain a mission impossible. The repertoire of the required compliant and compatible natural and artificial digit‐ and limb‐regeneration materials, like skin, cartilage, bone, blood vessel, nerve, tendon and ligament, and muscle, are all in various stages of developmental maturity. How to integrate all of them will be the last and perhaps the largest problem left to solve. Nevertheless, the biotechnical revolution has allowed us to turn many of the dreams of science fiction into current biomedical realities.

A summary of strategies and challenges for regeneration and repair of human digits and limbs is shown in Figure 10. Some are fact and some still fiction. The International Research Center for Wound Repair and Regeneration (iWRR) at National Cheng Kung University, Tainan, Taiwan, held an International Symposium on Wound Regeneration and Repair on 8 October 2013 (Fig. 11). Internationally renowned professors in the field of regenerative medicine—Cheng‐Ming Chuong (University of Southern California), David Gardiner (University of California, Irvine), Ken Muneoka (Tulane University), Malcolm Maden (University of Florida), Jonathan Slack (University of Minnesota), Randall Widelitz (University of Southern California), Shigeru Kondo (Osaka University), John Foley (Indiana University), Tai‐Lan Tuan (University of Southern California), Ting‐Xin Jiang (University of Southern California)—and many local faculty were invited to attend. The interesting issue of digit and limb regeneration and repair was raised, and a heated debate about the blastema and digit and limb regeneration ensued. Although it is obvious that many extremely difficult and perhaps unresolvable challenges exist for human digit and limb regeneration, we all expressed our hopes of one day translating the basic scientific research into clinical reality.
